# Using Deep Convolutional Neural Networks for Image-Based Diagnosis of Nutrient Deficiencies in Rice

**DOI:** 10.1155/2020/7307252

**Published:** 2020-09-09

**Authors:** Zhe Xu, Xi Guo, Anfan Zhu, Xiaolin He, Xiaomin Zhao, Yi Han, Roshan Subedi

**Affiliations:** ^1^College of Forestry, Jiangxi Agricultural University, Nanchang 330045, China; ^2^Key Laboratory of Poyang Lake Watershed Agricultural Resources and Ecology of Jiangxi Province, Jiangxi Agricultural University, Nanchang 330045, China; ^3^Soil and Fertilization Technology Extension Station of Jiangxi Province, Nanchang 330045, China; ^4^Institute of Agriculture and Animal Science, Tribhuvan University, Kathmandu 00977, Nepal

## Abstract

Symptoms of nutrient deficiencies in rice plants often appear on the leaves. The leaf color and shape, therefore, can be used to diagnose nutrient deficiencies in rice. Image classification is an efficient and fast approach for this diagnosis task. Deep convolutional neural networks (DCNNs) have been proven to be effective in image classification, but their use to identify nutrient deficiencies in rice has received little attention. In the present study, we explore the accuracy of different DCNNs for diagnosis of nutrient deficiencies in rice. A total of 1818 photographs of plant leaves were obtained via hydroponic experiments to cover full nutrition and 10 classes of nutrient deficiencies. The photographs were divided into training, validation, and test sets in a 3 : 1 : 1 ratio. Fine-tuning was performed to evaluate four state-of-the-art DCNNs: Inception-v3, ResNet with 50 layers, NasNet-Large, and DenseNet with 121 layers. All the DCNNs obtained validation and test accuracies of over 90%, with DenseNet121 performing best (validation accuracy = 98.62 ± 0.57%; test accuracy = 97.44 ± 0.57%). The performance of the DCNNs was validated by comparison to color feature with support vector machine and histogram of oriented gradient with support vector machine. This study demonstrates that DCNNs provide an effective approach to diagnose nutrient deficiencies in rice.

## 1. Introduction

Fertilizers are essential to global food production, particularly by ensuring high and stable yields of rice [1]. The best results come when specific fertilizers are applied in the needed amounts at the proper time. However, rice is often cultivated without such targeted nutrient input in China. Unscientific fertilization practices are common and, when coupled with a general delay between research findings and widespread adoption of technology, result in imbalanced nutrient application to rice fields. At present, blind fertilization still occurs frequently. As a result, ever greater amounts of fertilizers are applied to achieve only limited increases in rice yield, and the quality of the resulting rice declines [2]. This leads to smallholder farmers—who produce most of China's rice—not realizing potentially attainable increases in income.

Diagnosis of nutrient deficiencies in rice is an integral part of scientific fertilization, because soils often fail to completely meet the nutrient demands of growing plants. Determination of the needed nutrients will facilitate the formulation of a fertilization regime to supply the target nutrients without oversupplying others. Symptoms of a nutrient deficiency in rice are manifestations of malnutrition in the crop; they can be visually inspected from the plant morphology and thereby provide enough information to diagnose the nutrient deficiency [3]. Li et al. [4] analyzed the symptoms of silicon deficiency in rice and proposed its control measures. Problems arise as agricultural production sites are extensive, and many nutrient deficiencies are widely distributed both across a farm or region and in time throughout the growing season. It is therefore difficult for agricultural experts to meet the current demand for their services, which prevents the nutrient deficiencies from being properly diagnosed and effectively corrected.

In addition to manual diagnosis, chemical and nondestructive analytical methods have been used to identify nutrient deficiencies in crops. Chemical diagnoses can be classified as either full-scale analysis or rapid tissue measurement [5]. Full-scale analysis measures the contents of nutrient elements in a crop. This diagnostic technique can determine all the nutrient elements essential to a plant's survival and those involved in its growth [6]. The results are precise and reliable and usually provide sufficient data for diagnosis, but the technique involves vast labor costs and is confined to a laboratory. The rapid measurement of unassimilated nutrients in plant tissues is carried out by visual colorimetry, which is fast, straightforward, and generally suitable for field diagnosis [7]. Due to the extensive examination required for deeper analysis, this technique is usually applied to assess deficiencies of major elements such as N, P, and K in crops. The minimal contents of trace elements and the high precision required for their analysis prevent rapid measurement of their status.

Nondestructive diagnosis techniques include *in situ* measurement using a chlorophyll meter, spectral remote sensing, and computer vision. The first two of these methods are costly or complicated in data processing and are suitable only for specific elements [8, 9]. In contrast, computer vision requires only a smartphone for low-cost image acquisition and transfer, making image-based diagnosis potentially the best and most versatile solution. Conventional computer vision methods such as support vector machines (SVMs) have demonstrated their ability to detect nutrient deficiencies of five elements such as N, P, and K in the leaves of rape [10] and rice [11–14]; yet, SVMs run the risk of overfitting large datasets as they are sensitive to outliers. Convolutional neural networks (CNNs) are attractive machine learning tools [15], and since the success of deep CNNs (DCNNs) in the ImageNet Large Scale Visual Recognition Challenge 2012 (ILSVRC 2012), they have become the preferred choice for image recognition. However, the use of DCNNs to identify nutrient deficiencies in rice has rarely been reported [16].

Inspired by the Plant Village Project [17], we explore the accuracy of diagnosing nutrient deficiencies in rice by processing image data from hydroponic experiments, with four different DCNNs as potentially practical solutions for real-time crop assessment.

## 2. Related Work

Many studies have considered their use in diagnosis of crop diseases. For example, Mohanty et al. [18] trained a DCNN with deep learning using 54,306 images of healthy and infected plant leaves obtained from the Plant Village dataset to identify 14 crop species and 26 diseases. The study evaluated the feasibility of DCNNs for the diagnosis of crop diseases by using two architectures, AlexNet [19] and GoogLeNet [20], which achieved up to 99.4% accuracy. When using other labeled images taken under different conditions, the accuracy of the trained model was reduced substantially to 31.4%, which nonetheless was much higher than that based on random selection (2.6%). The deep learning model was therefore highly accurate but not robust. The Plant Village dataset based on expert knowledge might include some noise via variations in different experts' manual labeling, with images acquired in a similar background via a regularized process. This could account for the limitation in generating a robust model. As visible symptoms are time-dependent and tissue-specific, the dataset should be as extensive as possible. Follow-up work by Mohanty et al. [18] found that increasing the variety of the images would lead to better diagnosis of crop diseases.

Brahimi et al. [21] trained AlexNet and GoogLeNet on their own dataset and obtained 99% accuracy with transfer learning when identifying nine classes of tomato diseases. Their method was demonstrated to be more accurate than SVM and random forest. Based on AlexNet, Liu et al. [22] recognized four classes of diseases on apple leaves with up to 97.6% accuracy, while Lu et al. [23] sorted rice among ten disease classes with 95.5% accuracy. Moreover, Zhang et al. [24] detected nine classes of maize leaf diseases using improved GoogLeNet with an accuracy of 98.9%. Furthermore, Amara et al. [25] used the LeNet5 architecture [26] to distinguish three classes of banana diseases, achieving accuracies of 92.9%–98.6%.

All these studies have achieved high classification accuracy of crop diseases, demonstrating the feasibility of DCNNs in image-based diagnosis. A DCNN approach involves both data and algorithm factors. Regarding data, building a truly comprehensive database is difficult. The visual characteristics of the symptoms may change with the progression of crop diseases or nutrient deficiencies, and they can also depend on environmental factors such as humidity and temperature [17]. Therefore, a large number of photographs are needed to cover the entire range of possibilities. A further consideration is that all images need to be labeled correctly, which is usually labor-intensive and error-prone [27]. Uneven sampling can skew the optimization direction of the model in the wrong direction. To solve this issue, researchers have proposed weighted cross-entropy loss [28–30].

Regarding algorithms, since AlexNet [19] was the best-performed model at ILSVRC 2012, many studies have sought to improve its performance. A typical trend in the evolution of architectures is that the networks are getting deeper [31]. For example, ResNet [32] which won ILSVRC 2015 is approximately 20 times deeper than AlexNet and 8 times deeper than VGGNet [33]. In addition, GoogLeNet [20], the champion of ILSVRC 2014, proposes an inception structure to improve the utilization of network computing resources and increase the network breadth and depth for a constant amount of computation. Furthermore, DenseNet [34] improves network performance stereotypes without requiring deepening (like ResNet) or widening (like GoogLeNet) of the network structure.

From the perspective of image features, the feature reuse and substitution (bypass) settings can greatly reduce the network parameter number while alleviating the problem of gradient disappearance to some extent. Edna et al. [35] used the Plant Village dataset to fine-tune DenseNet121, which achieved a better accuracy than ResNet50, ResNet101, ResNet152, VGG16, and Inception-v4 [36]. Excluding the artificially designed architecture, Zoph et al. [37] constructed a NasNet model with two AutoML-designed modules and achieved a prediction accuracy of 82.7% on ImageNet's validation set.

Considering rice, Lu et al. [23] applied AlexNet to identify ten classes of plant diseases in this crop. Compared with the new state-of-the-art DCNNs, AlexNet is a relatively shallow CNN model, and diagnosis of plant nutrient deficiencies is another application scenario. Based on parallelized shallow CNNs, Watchareeruetai et al. [38] achieved the overall precision of 43.02%, recall of 52.13, and F-measure of 47.14 for identifying six classes of nutrient deficiencies in black gram leaves. Since the dataset contains various deficiency periods of plant leaves, it is difficult to classify different types of nutrient deficiencies. Recently, Sethy et al. [39] applied pretrained DCNNs with an SVM classifier to identify four levels of nitrogen deficiency in rice and achieved an accuracy of 99.8%. This encourages us to explore the ability of DCNNs to classify more elements with different deficiency phases in rice.

## 3. Materials and Methods

### 3.1. Hydroponic Experiment

The dataset is the key to fitting a model with good generalization ability. Many studies have used the Plant Village dataset, which is based on expert knowledge as some of the observed phenotypes and diseases can be straightforwardly identified by visual cues. Although the symptoms of nutrient deficiencies in rice are similar universally, a challenge remains in collecting images in the field covering all the deficiency types. Here, we designed a hydroponic experiment to collect images for 10 classes of nutrient deficiencies (N, P, K, Ca, Mg, S, Fe, Mn, Zn, and Si, each denoted by a minus sign followed by its chemical symbol, e.g., –N) and contrast them with rice plants under full nutrition, making a total of 11 classes.

**Figure 1:**
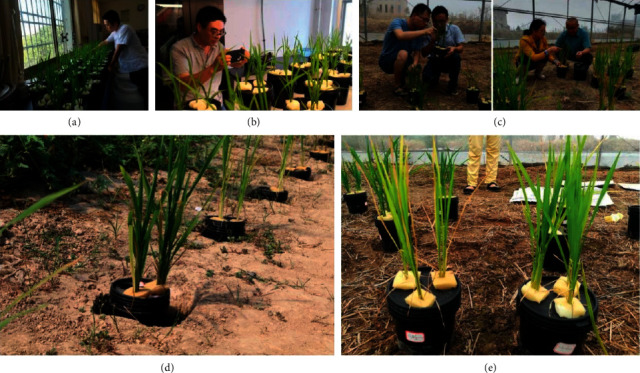
Hydroponic experiment and ground truth. From left to right: seedling preparation, image capture in the laboratory, symptom observation in the greenhouse (top), and ground truth of the experiment in 2017 and 2018 (bottom).

The experiment lasted for two years (2017–2018) and used late rice (*Oryza sativa L*.) “Taiyou 398,” the major local variety in Jiangxi Province, China. We designed the experiment as a single-factor test with five replications, giving a total of 55 treatments. Eleven nutrient solutions (pH 5.0–5.5) were prepared separately. The contents of full-strength Kimura B nutrient solution and 10 deficiency solutions are listed in Table 1. Each treatment was performed in a 2-L plastic bucket with a lid, and three holes were punched in the lid to hold rice seedlings. A single three-day-old seedling was planted in each hole, and the stem was wrapped with a sponge. After three days of adaptive cultivation in the laboratory, the plants were moved to a greenhouse to prevent contamination of the nutrient solution (Figure 1). During the growing period, the nutrient solution was changed every three days, and images of rice plants (especially the leaves) were collected.

Kimura B was used as a standard for full nutrition. Data in italic or left blank indicate deficient compounds. Deficiencies were created by substituting the compounds containing that nutrient element with other compounds to provide all the other nutrients except the selected deficient nutrient.

### 3.2. Dataset

The symptoms of nutrient deficiencies manifesting during the hydroponic experiments are recorded in Table 2. The morphological symptoms of rice plants differed among the 11 classes due to the nutrients having various physiological functions in the plant system. For example, Ca, Mg, Fe, Mn, and Zn are directly or indirectly related to chlorophyll formation and/or photosynthesis [40–42], and therefore their deficiencies result in chlorosis. As P is associated with carbohydrate metabolism [43, 44], its deficiency results in carbohydrate retention in the leaves and thus purple-red coloration developing in the stems and leaves due to the formation of anthocyanins.

The symptoms of nutrient deficiencies were also influenced by the mobility of the nutrients in the plant system. Mobile elements such as N, P, K, and Mg, when deficient, tend to move quickly toward the younger parts of the plant, making symptoms always appear first in older parts such as the lower leaves. Conversely, deficiencies of less mobile elements such as Ca and Fe often manifest in the younger parts of the plant.

**Figure 2:**
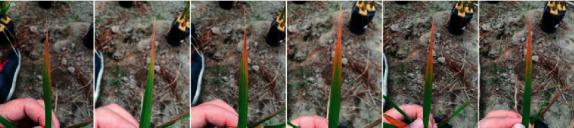
Symptoms of P nutrient deficiency on rice leaves photographed from different angles.

Mobile telephones or cameras captured images from plants leaves at different time points depending on the location and progress of the symptoms. Before photography, the deficiency symptom must be at least minimally recognizable, and the minimum recognized unit of the symptom was used as the image framing center. Photographs were taken from six different angles (Figure 2) and then cropped to show only the leaf.

After the development of typical deficiency symptoms (Table 2), we removed rice plants from the plastic bucket. The whole plants were washed with water and then deactivated at 110°C for 15 min. The materials were oven-dried at 75°C to constant weight before being crushed and analyzed for nutrient content in the laboratory.  Table 3 lists the standards and test methods followed to analyze each nutrient's content.

The images of rice leaves included complex backgrounds; they were given one of 11 class labels according to the assigned nutrient deficiency. The images were cropped to ensure that the symptoms appeared prominently and therefore reduce memory usage for better calculation performance. Because the time points of different deficiency symptoms are inconsistent, data collection cannot guarantee the balance of each class throughout the experimental period.  Table 4 gives details of the 1818 images created, 60% of which were used for training, 20% for validation, and 20% for test.

### 3.3. State-of-the-Art DCNNs

GoogLeNet [20] introduces the “Inception” concept, which changes a full connection to a sparse connection. It increases the breadth and depth of the network and eventually allows the utilization of network computing resources. The Inception module convolves the input simultaneously with convolution kernels of different sizes, plus a pooling operation, and finally aggregates the respective results together as a total output. The convolutions are of varied sizes (1 × 1, 3 × 3, and 5 × 5) for ease of alignment. As the network goes deeper, the features become increasingly abstract, and the receptive field involved in each feature becomes larger. Therefore, as the number of layers increases, the proportion (i.e., the amount) of 3 × 3 and 5 × 5 convolutions also increases. In this case, the number of parameters and the amount of calculations remain very large. The Inception module uses a bottleneck layer comprising a 1 × 1 convolution to help reduce the computation requirements. One of the most important improvements in Inception-v3 is the factorization of *n* × *n* convolution kernels into 1 × *n* and *n* × 1 convolutions, which speeds up computations and deepens the network.

ResNet [50, 51] solves the vanishing gradient problem in deeper networks using shortcut connections where the gradient reaches earlier layers and compositions of features at varying depth can be combined to improve performance. ResNet relies on many stacked residual units that are composed of convolution and pooling layers; it therefore acts as the building block to construct the network. The structure of ResNet consists of residual blocks, each of which is concatenated by three convolutions of 1 × 1, 3 × 3, and 1 × 1 kernels. The residual blocks are then converged by average pooling and classified using the softmax function.

DenseNet [34] provides an extreme example of attempting to overcome the training difficulties in deeper networks by introducing shortcut connections. It concatenates all previous layers to form the input of each layer, connecting each layer to all previous ones. DenseNet is designed from the perspective of image features; its feature reuse and bypass settings allow DenseNet to achieve state-of-the-art performance with fewer parameters than ResNet. ResNets and DenseNets achieve similar accuracy to visual geometry group (VGG) on the ImageNet dataset at only 20% and 10%, respectively, of its computational cost [52]. DenseNet enhances the transfer of gradients, facilitates feature reuse, and reduces overfitting of small sample data. Its structure consists of dense blocks linked by transition layers. Each dense block is constructed by the following steps: batch normalization, ReLU activation, convolution operation, batch normalization, ReLU activation, convolution operation, and final concatenation with the last input. Each transition block is constructed by batch normalization, ReLU activation, convolution operation, and average pooling operation.

NasNet [37], a new search space designed by Google, uses reinforcement learning to optimize the network structure in experiments. The structure of NasNet is similar to those of ResNet and Inception, and it performs basic block stacking to generate the final network. The network structure contains two main modules, a normal cell and a reduction cell, which are stacked to form the final network. NasNet-Large has been trained on the ImageNet database, and the network has learned rich feature representations for a wide range of images, with an image input size of 331 × 331.

### 3.4. Fine-Tuning

Fine-tuning is a technique in which knowledge is acquired during training for use in other related tasks or areas [53]. In a DCNN, the pretrained weight is trained to identify characteristics, some of which can be used in other target jobs. Therefore, during the learning process, the last layers of the trained network can be deleted and new layers can be retrained for the target job. Application of fine-tuning learning in experiments requires some learning, but the technique remains much quicker than learning from scratch [18]. In addition, fine-tuning is more accurate than models trained from scratch [35]. Herein, we applied all the DCNNs introduced in Section 3.3 with ImageNet pretraining weight and removed the top layer. The average pooling layer for each model was then added, followed by a fully connected layer with 1024 nodes. Finally, the 11 categories were classified using the softmax function. All the added layers were initialized with random parameters.

### 3.5. Weighted Categorical Cross-Entropy

Unevenly distributed images will lead to bias if one class has much more samples than the others. Therefore, weighted categorical cross-entropy is introduced to solve this problem. This method works by weighting the loss function so that the training process will focus more on the samples from an underrepresented class [30]. Its implementation is available at Keras (https://keras.io). The weighting value for each class is set to the reciprocal of the number of samples for that class.

## 4. Results and Discussion

### 4.1. DCNN Experiments

The experiments were performed on a Windows10 desktop equipped with one Intel Core i9 7920X CPU with 64 GB RAM, accelerated by two GeForce GTX 1080Ti GPUs with 11 GB memory. The model implementation was powered by the Keras framework with the TensorFlow backend. Fine-tuning models with weights pretrained on ImageNet were used for model fitting. No data augmentation was used. Evaluation of the models was performed by using the accuracy metric and kappa score. Model performance was evaluated three times, expressed as the average accuracy of training and validation, and graphically depicted for each model. The Adam optimizer was used to accelerate the training process. Images were resized through Python Image Library to a specific size based on the model requirement.

Repeated experiments comparing different learning rates (0.001, 0.0001, 0.00006, 0.00003, and 0.00001) showed that the learning rate of 0.00001 was most suitable for the model to run 50 epochs for training, and no decay was used. Due to GPU constraint with NasNet-Large, batch size cannot exceed 10. Therefore, to evaluate the models in terms of time efficiency, we set the same learning rate (0.00001) and batch size (10) for all the tests.  Table 5 lists the experiment details.

In addition, we compared the four DCNN models tested in this study with two traditional machine learning methods, color feature with SVM and HOG (Histogram of Oriented Gradient) with SVM [39, 54]. The color feature was read from the images directly and then trained with an SVM classifier (implementation was based on scikit-learn library); the HOG extraction process can be divided into 5 parts: detection window, normalized image, calculated gradient, statistical histogram and normalized gradient histogram, and obtained HOG feature vector. These steps are integrated with the hog.compute function of OpenCV-Python library.

### 4.2. Approach Analysis

**Figure 3:**
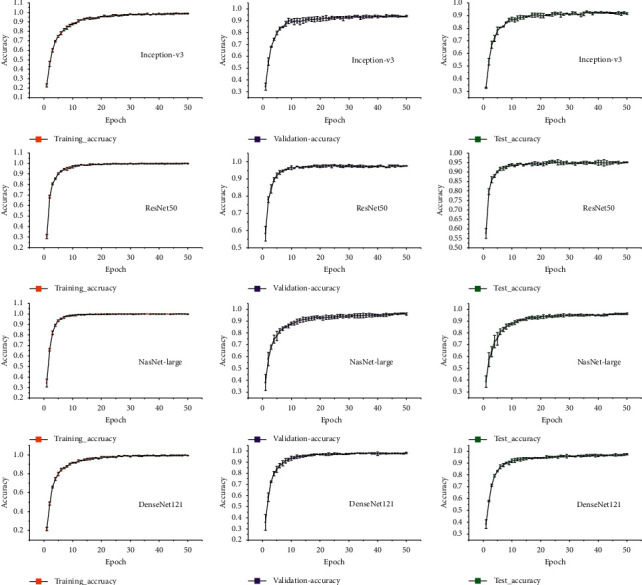
Average accuracy of four deep convolutional neural networks for prediction of nutrient deficiencies in rice.

This study used four state-of-the-art DCNNs to evaluate the performance of image recognition techniques in identifying nutrient deficiencies in rice. All the four DCNNs, Inception-v3, ResNet with 50 layers, NasNet-large, and DenseNet with 121 layers, were effectively fitted (Figure 3). After fine-tuning, all the models achieved average training, validation, and test accuracies of over 90% and yielded average kappa scores of over 0.90, which were much higher than those of color feature with SVM and HOG with SVM (Table 5). Except for Inception-v3, the remaining three models achieved over 95% average accuracies. Specifically, ResNet50 and NasNet-Large obtained similar prediction accuracies, while DenseNet121 required fewer parameters and attained a slightly higher accuracy than the other models. The test and validation kappa scores of DenseNet121 were over 0.97, and its time efficiency was acceptable.

From the perspective of approach process, NasNet-Large showed a slow increase in its test and validation accuracies, which might be due to the huge number of parameters in this model compared with other models. Thus, during backpropagation, the pretrained parameters of NasNet-Large were updated slowly to obtain good generalization ability. Moreover, the DCNNs were able to adequately fit the data, while the two conventional methods (i.e., color feature with SVM and HOG with SVM) seemed to give an overfitting. The possible reason is that some of the labeled images had complex background where the outline data would easily lead to overfitting in the SVM approach. In contrast, the DCNN approach can synthesize local receptive field in higher-level networks.

### 4.3. Qualitative Analysis

Since DenseNet121 performed the best, one run of this model was used for qualitative analysis. Three indices were calculated to gain a better understanding of the model's prediction accuracy. The recall refers to the ratio of correctly predicted positive observations to the all observations in an actual class. The precision refers to the ratio of correctly predicted positive observations to the total predicted positive observations. The f1-score refers to the weighted average of precision and recall, which therefore considers both false positives and false negatives; f1 is usually more useful, especially for an uneven class distribution, albeit not as intuitively understandable as accuracy.  Table 6 gives the good prediction results for –N, –P, –K, –Ca, –S, –Mn, –Zn, and –Si, with f1-scores of over 0.95, while performance regarding –Fe was the worst. The relative small number of samples in the –Fe case (*n* = 31) might account for the poor predictive performance.

**Figure 4:**
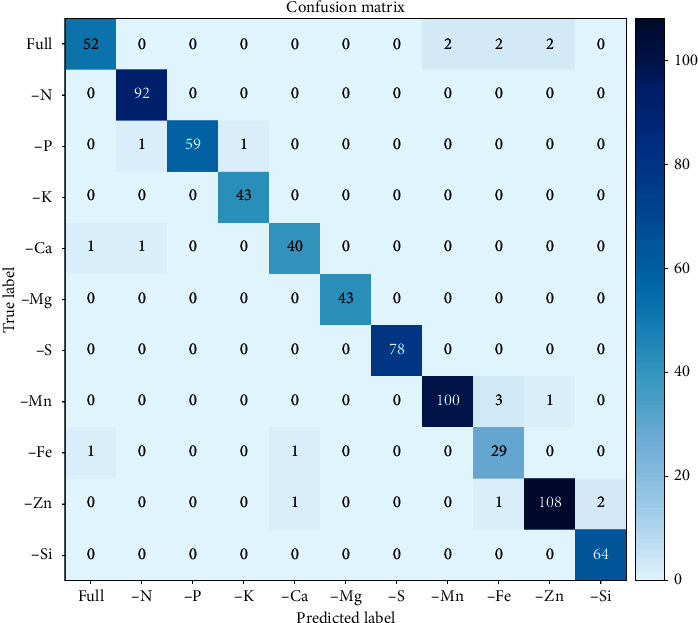
Confusion matrix for DenseNet121 in prediction of nutrient deficiencies in rice.

Deficiency is a relative concept, and a slight deficiency might be mistaken for full nutrition. In this study, another area for misclassification was among Fe, Mn, and Zn deficiencies which all shared some common symptoms. These nutrients are directly or indirectly related to chlorophyll formation or photosynthesis, the disruption of which generally causes chlorosis [38]. Furthermore, Zn deficiency was misclassified as Si deficiency as both of them caused brown spots in rice leaves. These noticeable values can be extracted from the confusion matrix (Figure 4).

### 4.4. Application Analysis

DCNNs demonstrate powerful capabilities in image recognition. Open source development frameworks lower the barriers to the application, as their versatile platform portability greatly facilitates the progression from concepts and plans to results and applications. New vision-problem applications can often use the architectures of networks already published in the literature alongside open source implementation to ease development, as prior studies might have solved various detailed technical problems such as the learning rate decay schedule or the hyperparameters. Optimizing hyperparameter settings is a significant challenge owing to the enormous time cost. Automatic model design represents a good solution as increasing numbers of applications are developed and shared [55].

Although some DCNNs can rescale image size, it is necessary to pay attention to the spatial resolution conditions of the rice leaf images. If the region of the identified object is too small in the overall image, the recognition accuracy will be affected. In addition, the background image can affect the recognition accuracy, especially in cases of multiclass identification. Unlike image recognition tasks involving the identification of people or car objects, the identification of nutrient deficiencies in rice leaves involves discerning subtle differences of texture in often similar images. Recognizing nutritional deficiencies in rice should consider surface texture as an identification feature, because this study aims to identify multiple images of nutrition deficiencies in rice. At the beginning, we used the original image (resolution 2976 × 3968), but, owing to the GPU memory constraint, we resized it to 600 × 800 and used a shallow CNN for the experiment. The result had low accuracy. In a subsequent experiment, cropping the image to an individual leaf effectively improved the recognition accuracy; therefore, any application of this technique needs to combine region-based CNN for the automatic extraction of the leaf parts in the images and thereby reduce the influence of the variable image backgrounds.

Compared with other datasets that are based on expert knowledge, the symptoms manifesting in the hydroponic experiments would be more precise, because other factors such as pests and diseases are eliminated under controlled conditions [27], and the data cover the entire rice growing period. However, we did not consider combinations of more than one deficiency factor in this study, which reduced the robustness of the model. Hydroponic experiments represent a time- and resource-intense means of data collecting to build a model with generalized ability. Images alone are always insufficient for supervised classification, so any application should employ data augmentation techniques such as cropping, rotating, and flipping before running recognition processes [56]. The application side should include a label function that uploads the confirmed prediction result to augment the dataset. As similar symptoms can lead to misclassification, offering the top-three predictions would be better than providing only one. Moreover, the symptoms associated with insect-pests and diseases are similar to the visual characteristics of nutrient deficiencies [57]; thus, combined studies are needed to gain better insight into the identification of practical problems.

This study in the short term will optimize the models and datasets for diagnosis of nutrient deficiencies in rice. It can then be integrated into a mobile diagnosis system to facilitate rice production by smallholder farmers. In the long run, if a model with greater generalization ability is proposed, the permissions of the mobile users to upload results with locations would provide a vast dataset to aid digital soil mapping and fertilization assessment from a macro perspective.

## 5. Conclusion

Different nutrient deficiencies alter the morphological characteristics of plant leaves in rice. In this study, four DCNNs, Inception-v3, ResNet50, NasNet-Large, and DenseNet121, were used to diagnose various nutrient deficiencies in rice plants based on image recognition using a dataset collected from hydroponic experiments. All the DCNNs obtained accuracies of over 90% and outperformed two traditional machine learning methods, color feature with SVM and HOG with SVM. The best result was obtained using DenseNet121 with the validation accuracy of 98.62% and test accuracy of 97.44%. These findings demonstrate that DCNNs are promising for fully automatic classification of nutrient deficiencies in rice.

Future work should collect more outdoor images and design field experiments to make a more extensive dataset and implement the region-based CNN object detection module to extract rice leaf images for diagnosis in practice. Conducting further hydroponic experiments to build an image dataset covering multiple deficiency factors could improve the models for better diagnosis of multinutrient deficiencies.

## Figures and Tables

**Table 1:** Solution contents for each class of nutrient deficiency in rice (mM = mmol/L; *μ*M = *μ*mol/L).

Class	Conc.											
	0.37 mM	0.55 mM	0.18 mM	0.37 mM	0.21 mM	20 *μ*M	6.7 *μ*M	0.105 *μ*M	0.15 *μ*M	0.16 *μ*M	9.4 *μ*M	1.5 mM
Full	(NH_4_)_2_ SO_4_	MgSO_4_·7H_2_O	KNO_3_	Ca(NO_3_)_2_·4H_2_O	KH_2_PO_4_	*FeCl*_*2*_ *-EDTA*	MnCl_2_·4H_2_O	Na_2_MoO_4_·2H_2_O	ZnSO_4_·7H_2_O	CuSO_4_·5H_2_O	H_3_BO_3_	Na_2_SiO_3_·9H_2_O
−N	*Na*_*2*_ *SO*_*4*_	MgSO_4_·7H_2_O	*KCl*	*CaCl*_*2*_	KH_2_PO_4_	*FeCl*_*2*_ *-EDTA*	MnCl_2_·4H_2_O	Na_2_MoO_4_·2H_2_O	ZnSO_4_·7H_2_O	CuSO_4_·5H_2_O	H_3_BO_3_	Na_2_SiO_3_·9H_2_O
−P	(NH_4_)_2_ SO_4_	MgSO_4_·7H_2_O	KNO_3_	Ca (NO_3_)2·4H_2_O	*KCl*	*FeCl*_*2*_ *-EDTA*	MnCl_2_·4H_2_O	Na_2_MoO_4_·2H_2_O	ZnSO_4_·7H_2_O	CuSO_4_·5H_2_O	H_3_BO_3_	Na_2_SiO_3_·9H_2_O
−K	(NH_4_)_2_ SO_4_	MgSO_4_·7H_2_O	*NaNO*_*3*_	Ca (NO_3_)_2_·4H_2_O	*Na*_*2*_ *HPO*_*4*_	*FeCl*_*2*_ *-EDTA*	MnCl_2_·4H_2_O	Na_2_MoO_4_·2H_2_O	ZnSO_4_·7H_2_O	CuSO_4_·5H_2_O	H_3_BO_3_	Na_2_SiO_3_·9H_2_O
−Ca	(NH_4_)_2_ SO_4_	MgSO_4_·7H_2_O	KNO_3_	*NaNO*_*3*_ *(0.74 mM)*	KH_2_PO_4_	*FeCl*_*2*_ *-EDTA*	MnCl_2_·4H_2_O	Na_2_MoO_4_·2H_2_O	ZnSO_4_·7H_2_O	CuSO_4_·5H_2_O	H_3_BO_3_	Na_2_SiO_3_·9H_2_O
−Mg	(NH_4_)_2_ SO_4_	*Na*_*2*_ *SO*_*4*_	KNO_3_	Ca(NO_3_)_2_·4H_2_O	KH_2_PO_4_	*FeCl*_*2*_ *-EDTA*	MnCl_2_·4H_2_O	Na_2_MoO_4_·2H_2_O	ZnSO_4_·7H_2_O	CuSO_4_·5H_2_O	H_3_BO_3_	Na_2_SiO_3_·9H_2_O
−S	*NH*_*4*_ *Cl (0.74 mM)*	*MgCl*_*2*_	KNO_3_	Ca(NO_3_)_2_·4H_2_O	KH_2_PO_4_	*FeCl*_*2*_ *-EDTA*	MnCl_2_·4H_2_O	Na_2_MoO_4_·2H_2_O	*ZnCl*_*2*_	*CuCl*_*2*_	H_3_BO_3_	Na_2_SiO_3_·9H_2_O
−Mn	(NH_4_)_2_ SO_4_	MgSO_4_·7H_2_O	KNO_3_	Ca(NO_3_)_2_·4H_2_O	KH_2_PO_4_	*FeCl*_*2*_ *-EDTA*	*NaCl (13.4 μM)*	Na_2_MoO_4_·2H_2_O	ZnSO_4_·7H_2_O	CuSO_4_·5H_2_O	H_3_BO_3_	Na_2_SiO_3_·9H_2_O
−Fe	(NH_4_)_2_ SO_4_	MgSO_4_·7H_2_O	KNO_3_	Ca(NO_3_)_2_·4H_2_O	KH_2_PO_4_		MnCl_2_·4H_2_O	Na_2_MoO_4_·2H_2_O	ZnSO_4_·7H_2_O	CuSO_4_·5H_2_O	H_3_BO_3_	Na_2_SiO_3_·9H_2_O
−Zn	(NH_4_)_2_ SO_4_	MgSO_4_·7H_2_O	KNO_3_	Ca(NO_3_)_2_·4H_2_O	KH_2_PO_4_	*FeCl*_*2*_ *-EDTA*	MnCl_2_·4H_2_O	Na_2_MoO_4_·2H_2_O	*Na*_*2*_ *SO*_*4*_	CuSO_4_·5H_2_O	H_3_BO_3_	Na_2_SiO_3_·9H_2_O
−Si	(NH_4_)_2_ SO_4_	MgSO_4_·7H_2_O	KNO_3_	Ca(NO_3_)_2_·4H_2_O	KH_2_PO_4_	*FeCl*_*2*_ *-EDTA*	MnCl_2_·4H_2_O	H_24_Mo_7_N_6_O_24_·4H_2_O	ZnSO_4_·7H_2_O	CuSO_4_·5H_2_O	H_3_BO_3_	

**Table 2:** Symptoms of nutrient deficiencies in rice plants throughout the growing period.

Class	Typical symptoms
Full	Leaves are green, generally with no mottling or spots.

−N	First appear in the lower leaves of the main stem and then gradually develop in the upper part. Leaves turn from green to yellow starting at the tip and extending along the midrib to the base of the leaf in a Λ shape until the whole leaf is chlorotic and yellow.

−P	First appear in the lower leaves of the main stem and then gradually develop in the upper part. Lower leaves are dark green, and then old leaves become yellow. In severe cases, the lower leaves are longitudinally curled with cyan or brown spots.

−K	First appear in old leaves and then gradually extend to new leaves, leaf tips, and leaf stems. Basal leaves gradually turn yellow or yellowish brown from the tip, along the edge, to the base of the leaf. Reddish brown or dark brown rust spots of varying sizes emerge. In severe cases, spots form plaques and spots appear on the sheath. Later, the tip of the leaf gradually turns red and brown, and the discoloration gradually spreads from the lower leaves to the upper leaves.

−Ca	Present on new and upper leaves. The tips of the fresh leaves turn white, curl, and shrink. In particularly severe cases, the growth point is necrotic. The upper leaf tip and leaf margin are yellow.

−Mg	First appear in the lower, old leaves, which become chlorotic, while the veins remain green with clear yellow-green stripes. Discoloration starts at the leaf tips and then extends to the middle and rear parts.

−S	First appear in young leaves, which turn yellow. In severe cases, old leaves turn yellow and even white. New leaves are green and yellow, and their tips are scorched. There are also water-soaked round brown spots.

−Mn	Manifest in middle and upper leaves, which become chlorotic and yellowish. The veins remain green, causing a great color difference between them and the rest of the leaves. Gray or white spots appear in severe cases.

−Fe	Appear only in new leaves, while old leaves remain healthy. Young leaves are chlorotic, yellow, and white, but the veins stay green.

−Zn	Appear in both new and old leaves. The bases of fresh leaves become chlorotic and white. Irregular brown spots appear on both sides of the middle and lower leaves. In severe cases, the brown spots extend to the leaf sheaths, which turn red and brown from the tip of the leaves, generally appearing from the lower leaves to the upper leaves.

−Si	Appear on lower leaves as brown spots.

**Table 3:** Standards and methods followed to analyze nutrient contents in rice plants.

Nutrient	Standard	Method	Reference
N	HJ 636-2012	Alkaline potassium persulfate digestion UV spectrophotometry	[45]
P	GB/T 11893-1989	Ammonium molybdate spectrophotometry	[46]
K, Ca, Mg, Mn, and Fe	GB 5009.268-2016	Inductively coupled plasma mass spectrometry	[47]
Zn	GB 5009.268-2016	Inductively coupled plasma mass spectrometry	[47, 48]
S and Si	HJ 776-2015	Inductively coupled plasma optical emission spectrometry	[49]

**Table 4:** Numbers of images for 11 classes of nutrient deficiencies.

Class	Images	Typical symptoms
Full	164	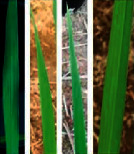
−N	246	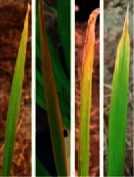
−P	154	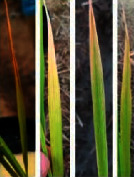
−K	108	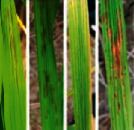
−Ca	111	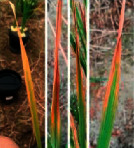
−Mg	101	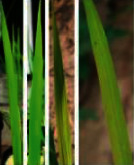
−S	208	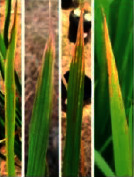
−Mn	230	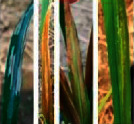
−Fe	74	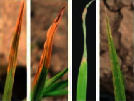
−Zn	246	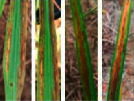
−Si	176	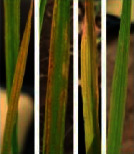

**Table 5:** Convolutional neural networks and specifications for experiments. Accuracy and kappa score of training, validation, and test are also included.

Model	Parameters (millions)	Layers	Training accuracy (%)	Validation accuracy (%)	Test accuracy (%)	Validation kappa score	Test kappa score	Training time per epoch (1090 samples)	Validation time per epoch (364 samples)
Inception-v3	23.9	48	98.75 ± 0.19	93.86 ± 0.57	91.67 ± 0.63	0.9314 ± 0.0064	0.9070 ± 0.0070	34 s	2 s
ResNet50	26.7	50	99.81 ± 0.14	97.53 ± 0.01	95.15 ± 0.16	0.9723 ± 0.0002	0.9478 ± 0.0022	30 s	2 s
NasNet-Large	84.9	—	99.79 ± 0.19	95.88 ± 0.95	96.25 ± 0.57	0.9539 ± 0.0106	0.9611 ± 0.0093	199 s	10 s
DenseNet121	8.1	121	99.30 ± 0.05	98.62 ± 0.57	97.44 ± 0.57	0.9794 ± 0.0064	0.9713 ± 0.0063	48 s	3 s
Color feature + SVM (RBF kernel)	—	—	90.55	66.48	64.01	0.6220	0.5951	—	—
HOG + SVM (RBF kernel)	—	—	93.76	56.93	56.86	0.5105	0.5064	—	—

**Table 6:** Recall, precision, and f1-score per class of nutrient deficiency for DenseNet121.

Class	Recall	Precision	f1	Samples
Full	0.96	0.90	0.93	58
−N	0.98	1.00	0.99	92
−P	1.00	0.97	0.98	61
−K	0.98	1.00	0.99	43
−Ca	0.95	0.95	0.95	42
−Mg	1.00	1.00	1.00	43
−S	1.00	1.00	1.00	78
−Mn	0.98	0.96	0.97	104
−Fe	0.83	0.94	0.88	31
−Zn	0.97	0.96	0.97	112
−Si	0.97	1.00	0.98	64

## Data Availability

The data used to support the findings of this study are available from the corresponding author upon request.

## References

[1] Xu X., He P., Yang F. (2017). Methodology of fertilizer recommendation based on yield response and agronomic efficiency for rice in China. *Field Crops Research*.

[2] Liu X., Zhang Y., Han W. (2013). Enhanced nitrogen deposition over China. *Nature*.

[3] Lu J. W., Li R. (2012). *Symptoms and Correction Techniques of Common Deficiency Syndrome in Rice*.

[4] Li Q., Cheng S., Chi S. J., Liu Y., Ni W., Pan S. (2014). Silicon-deficiency symptoms and its control measures in rice planting progress. *Modern Agricultural Science & Technology*.

[5] Ata-Ul-Karim S., Cao Q., Zhu Y., Tang L., Rehmani M. I. A., Cao W. (2016). Non-destructive assessment of plant nitrogen parameters using leaf chlorophyll measurements in rice. *Frontiers in Plant Science*.

[6] Xiong X., Zhang J., Guo D., Chang L., Huang D. (2019). Non-invasive sensing of nitrogen in plant using digital images and machine learning for Brassica Campestris ssp. Chinensis L. *Sensors*.

[7] Basavaraj S. A., Naveen N. M., Surendra P. (2020). Classification of yield affecting biotic and abiotic paddy crop stresses using field images. *Information Processing in Agriculture*.

[8] Balasubramanian V., Morales A. C. C., Cruz R. T. T., Thiyagarajan T. M., Nagarajan R., Abdulrachman S. (2000). Adaptation of the chlorophyll meter (SPAD) technology for real-time n management in rice: a review. *International Rice Research Notes*.

[9] Li J., Wan Y. J., Yan Y. Z., Ge S. (2017). Research progress on nondestructive rapid nutrition diagnosis of crop nitrogen. *China Rice*.

[10] Zhang K., Zhang A., Li C. S. (2016). Nutrient deficiency diagnosis method for rape leaves using color histogram on HSV space. *Transactions of the Chinese Society of Agricultural Engineering*.

[11] Chen L. S., Zhang S. J., Wang K., Shen Z. Q., Deng J. S. (2013). Identifying of rice phos-phorus stress based on machine vision technology. *Life Science Journal*.

[12] Chen L. S., Wang K. (2014). Diagnosing of rice nitrogen stress based on static scanning technology and image information extraction. *Journal of Soil Science and Plant Nutrition*.

[13] Chen L. S., Lin L., Cai G. (2014). Identification of Nitrogen, Phosphorus, and Potassium deficiencies in rice based on static scanning technology and hierarchical identification method. *PLoS One*.

[14] Chen L. S., Sun Y. Y., Wang K. (2017). Rapid diagnosis of nitrogen nutrition status in rice based on static scanning and extraction of leaf and sheath characteristics. *International Journal of Agricultural and Biological Engineering*.

[15] LeCun Y., Bengio Y., Hinton G. (2015). Deep learning. *Nature*.

[16] Barbedo J. G. A. (2019). Detection of nutrition deficiencies in plants using proximal images and machine learning: a review. *Computers and Electronics in Agriculture*.

[17] Hughes D. P., Salathe M. (2015). An open access repository of images on plant health to enable the development of mobile disease diagnostics. https://arxiv.org/abs/1511.08060.

[18] Mohanty S. P., Hughes D. P., Salathé M. (2016). Using deep learning for image-based plant disease detection. *Frontiers in Plant Science*.

[19] Krizhevsky A., Sutskever I., Geoffrey E. H. (2012). ImageNet classification with deep convolutional neural networks. *Advances in Neural Information Processing Systems*.

[20] Szegedy C., Liu W., Jia Y. Going deeper with convolutions.

[21] Brahimi M., Boukhalfa K., Moussaoui A. (2017). Deep learning for tomato diseases: classification and symptoms visualization. *Applied Artificial Intelligence*.

[22] Liu B., Zhang Y., He D., Li Y. (2017). Identification of apple leaf diseases based on deep convolutional neural networks. *Symmetry*.

[23] Lu Y., Yi S., Zeng N., Liu Y., Zhang Y. (2017). Identification of rice diseases using deep convolutional neural networks. *Neurocomputing*.

[24] Zhang X., Qiao Y., Meng F., Fan C., Zhang M. (2018). Identification of maize leaf diseases using improved deep convolutional neural networks. *IEEE Access*.

[25] Amara J., Bouaziz B., Algergawy A. A deep learning-based approach for banana leaf diseases Classification.

[26] Lecun Y., Bottou L., Bengio Y., Haffner P. (1998). Gradient-based learning applied to document recognition. *Proceedings of the IEEE*.

[27] Barbedo J. G. A. (2018). Factors influencing the use of deep learning for plant disease recognition. *Biosystems Engineering*.

[28] Ronneberger O., Fischer P., Brox T. (2015). U-net: convolutional networks for biomedical image segmentation. https://arxiv.org/abs/1505.04597.

[29] Cai Z., Fan Q., Feris R. S., Vasconcelos N. A unified multi-scale deep convolutional neural network for fast object detection.

[30] Aurelio Y. S., De Almeida G. M., De Castro C. L., Braga A. P. (2019). Learning from imbalanced data sets with weighted cross-entropy function. *Neural Processing Letters*.

[31] Gu J., Wang Z., Kuen J. (2017). Recent advances in convolutional neural networks. https://arxiv.org/abs/1512.07108.

[32] He K., Zhang X., Ren S., Sun J. Deep residual learning for image recognition.

[33] Simonyan K., Zisserman A. (2014). Very deep convolutional networks for large-scale image recognition. https://arxiv.org/abs/1409.1556.

[34] Huang G., Liu Z., M Laurens V. D., Weinberger K. Q. Densely connected convolutional networks.

[35] Too E. C., Yujian L., Njuki S., Yingchun L. (2019). A comparative study of fine-tuning deep learning models for plant disease identification. *Computers & Electronics in Agriculture*.

[36] Szegedy C., Vincent V., Sergey I., Jon S., Zbigniew W. Rethinking the inception architecture for computer vision.

[37] Zoph B., Vijay V., Jonathon S., Quoc V. L. (2017). Learning transferable architectures for scalable image recognition. https://arxiv.org/abs/1707.07012.

[38] Watchareeruetai U., Noinongyao P., Wattanapaiboonsuk C., Khantiviriya P., Duangsrisai S. Identification of plant nutrient deficiencies using convolutional neural networks.

[39] Sethy P. K., Barpanda N. K., Rath A. K., Behera S. K. (2020). Nitrogen deficiency prediction of rice crop based on convolutional neural network. *Journal of Ambient Intelligence and Humanized Computing*.

[40] Vatansever R., Ozyigit I. I., Filiz E. (2017). Essential and beneficial trace elements in plants, and their transport in roots: a review. *Applied Biochemistry and Biotechnology*.

[41] Merle T., Tavakol E., Bálin J. (2018). Functioning of potassium and magnesium in photosynthesis, photosynthate translocation and photoprotection. *Physiologia Plantarum*.

[42] Hochmal A. K., Schulze S., Trompelt K., Hippler M. (2015). Calcium-dependent regulation of photosynthesis. *Biochimica et Biophysica Acta (BBA)-Bioenergetics*.

[43] Gibson T. S. (1988). Carbohydrate metabolism and phosphorus/salinity interactions in wheat (*Triticum aestivum L.*). *Plant and Soil*.

[44] Jeong K., Julia C. C., Waters D. L. E. (2017). Remobilisation of phosphorus fractions in rice flag leaves during grain filling: implications for photosynthesis and grain yields. *PLoS One*.

[45] Zhu X. C., Zhou X. L., Lei X., Mao L. J. (2013). Discussion on new standard method HJ 636-2012 for determining total nitrogen. *China Water & Wastewater*.

[46] Lan W., Wu A., Wang Y., Wang J., Li J. (2019). Ionic solidification and size effect of hemihydrate phosphogypsum backfill. *China Environmental Science*.

[47] Xiong T., Wu Y., Li L. (2019). Material characteristics and eating quality of *Trachinotus ovatus* muscle. *Food Science*.

[48] Wei Y., Qiu S., Zhang J. Characteristic of heavy metal contents in agricultural wastes and agricultural risk assessment. *Transactions of the Chinese Society of Agricultural Engineering*.

[49] Sha D. R., Yang J. (2016). Determination of 32 elements in the industrial wastewater by ICP-OES. *Modern Scientific Instruments*.

[50] Hu H., Dey D., Del Giorno A., Hebert M., Bagnell J. A. (2018). Log-densenet: how to sparsify a densenet. https://arxiv.org/abs/1711.00002.

[51] He K., Zhang X., Ren S., Sun J. Identity mappings in deep residual networks.

[52] Huang G., Liu S., M Laurens V. D., Weinberger K. Q. (2017). Condensenet: an efficient densenet using learned group convolutions. https://arxiv.org/abs/1711.09224.

[53] Tan C., Sun F., Kong T., Zhang W., Yang C., Liu C. (2018). A survey on deep transfer learning. https://arxiv.org/abs/1808.01974.

[54] Dalal N., Triggs B. Histograms of oriented gradients for human detection.

[55] Pham H., Guan M. Y., Zoph B., Le Q. V., Dean J. (2018). Efficient neural architecture search via parameter sharing. https://arxiv.org/abs/1802.03268.

[56] Perez L., Wang J. (2017). The Effectiveness of data augmentation in image classification using deep learning. https://arxiv.org/abs/1712.04621.

[57] Barbedo J. G. A. (2016). A review on the main challenges in automatic plant disease identification based on visible range images. *Biosystems Engineering*.

